# Metabolically Abnormal But Normal-Weight Individuals Had a Higher Risk of Type 2 Diabetes Mellitus in a Cohort Study of a Chinese Population

**DOI:** 10.3389/fendo.2021.724873

**Published:** 2021-11-03

**Authors:** Qiannan Chen, Yaohan Zhou, Chen Dai, Gang Zhao, Yimin Zhu, Xuhui Zhang

**Affiliations:** ^1^ Basic Discipline of Chinese and Western Integrative, School of Public Health, Zhejiang Chinese Medical University, Hangzhou, China; ^2^ Department of Epidemiology & Biostatistics, School of Public Health, Zhejiang University, Hangzhou, China; ^3^ Department of Endocrinology and Institute of Cardiovascular Diseases, Putuo District People’s Hospital , Zhoushan, China; ^4^ Department of office Hangzhou Center of Disease Control and Prevention, Hangzhou, China; ^5^ Department of Respiratory Diseases, Sir Run Run Shaw Hospital Affiliated to the School of Medicine, Zhejiang University, Hangzhou, China; ^6^ Affiliated Hangzhou Center of Disease Control and Prevention, Zhejiang University School of Public Health, Hangzhou Center for Disease Control and Prevention, Hangzhou, China

**Keywords:** obesity, heterogeneity, type 2 diabetes mellitus, metabolically abnormal but normal weight (MANW), metabolically normal but obesity/overweight (MNO)

## Abstract

**Aims:**

Obesity is a heterogeneous disease in terms of body mass index (BMI) and metabolic status. The purpose of this study was to investigate the risk of type 2 diabetes mellitus (T2DM) in subjects with metabolically abnormal but normal weight (MANW) in China.

**Materials and Methods:**

A prospective cohort with a total of 17,238 participants of the Zhejiang metabolic syndrome cohort was recruited. According to the standard of the Working Group on Obesity in China, general obesity is defined. Metabolic abnormality was defined as two or more abnormal components (elevated triglycerides (TG), low high-density lipoprotein cholesterol (HDL-C), elevated systolic blood pressure (SBP) or diastolic blood pressure (DBP) or use of antihypertensive therapy, and elevated fasting plasma glucose or antidiabetic treatment). The hazard ratio (HR) and its 95% CI were calculated using a multiple regression model, adjusted for the potential confounding factors.

**Results:**

Compared with metabolically normal and normal weight (MNNW) subjects, the metabolically abnormal and obesity/overweight (MAO) subjects had the highest risk of T2DM disease, with an HR of 4.67 (95% CI: 3.23–6.76), followed by MANW subjects (HR = 2.61, 95% CI: 1.74–3.92) and metabolically normal but obesity/overweight (MNO) subjects (HR = 2.09, 95% CI: 1.29–3.38) after adjusting for age, sex, smoking, drinking, physical activity, and family history of diabetes. Compared with that in the MNNW subjects, the HR in MANW subjects was significantly higher than that in MNO subjects. In normal-weight subjects, the HR of T2DM was significantly positively correlated with the number of components with metabolic abnormalities.

**Conclusions:**

MANW subjects had a higher risk of T2DM. MANW subjects should be given more attention in the prevention and control of common chronic diseases.

## Introduction

Over the past few decades, the prevalence of overweight and obesity has risen rapidly around the world and has become a serious public health concern (1). Obesity increases the risks of cardiovascular disease, type 2 diabetes mellitus (T2DM), and all-cause mortality (2). Obesity is a heterogeneous disease with different phenotypes. According to the metabolic status, obesity can be divided into metabolic normality and abnormality obesity (3). Subjects who are obese without metabolic abnormalities are called metabolically normal but obesity/overweight (MNO) and account for about 18%–44%. About 5%–45% of individuals with normal weight have abnormal metabolic profiles (4, 5), which are known as metabolically abnormal but normal weight (MANW) population, and this group is relatively easy to ignore. Currently, many studies have been conducted on the relationship between MNO and the risk of developing diabetes, although the findings are inconsistent (6–8). However, there are relatively few studies on the risk of diabetes in MANW.

Previous studies have reported that MANW subjects might have an increased risk of long-term effects (8–11), but there was not enough research on the risk of diabetes in MANW. There is little reliable evidence from prospective studies with large sample size, especially in Chinese groups. Unlike Westerners, the Chinese had a lower body mass index (BMI) and smaller body size but had a higher body and visceral fat and a lower fat-free mass (12). In comparison with Caucasians, Chinese have more severe adverse profiles of metabolic components at the same BMI (13, 14). Therefore, it is of far-reaching significance to determine the relationship between different metabolic phenotypes and the risk of developing diabetes in a Chinese population, especially to determine the relationship between this MANW population that is not easy to pay attention to and diabetes.

In this study, we investigated the association between the heterogeneous phenotype of obesity and the risk of T2DM using a prospective cohort study of a total of 17,238 participants in China, and we tested the hypothesis that MANW individuals are at increased risk of diabetes.

## Materials and Methods

### Study Population

The subjects were recruited from the Zhejiang metabolic syndrome cohort in the Zhejiang Province, Southeastern China.

The cohort is a community-based prospective cohort that started from 2010 to 2014. This investigation was a study of 22,649 participants in five counties/communities in Zhejiang Province. The subjects were recruited from the residents of the cluster sample communities. This sample can represent the general population of the Zhejiang Province in terms of geographical location, demographic characteristics, and socioeconomic status. The epidemiological investigation, clinical health examination, and routine biochemical measurements were conducted at baseline. The protocols have been described in detail in the previous studies (1, 2, 15) and are briefly described below.

The participants were recruited for this study if they were ≥18 years old and had a BMI ≥18.5 kg/m^2^. Participants were excluded from baseline if they had cancer, diabetes, severe cardiovascular disease, or cerebrovascular disease (angina, cerebral infarction, and renal insufficiency) or if they had missing data for physical examination, such as weight, height, blood pressure (BP), waist circumference (WC), or biochemical determination, including triglycerides (TG), high-density lipoprotein cholesterol (HDL-C), and fasting plasma glucose (FPG). The participants who failed follow-up or migrated to other places (out of the county or city) were also excluded from the study. Ultimately, a total of 17,238 participants were recruited in the cohort.

The protocol was approved by the Ethics Committees of both Zhejiang University School of Medicine. Written informed consent was obtained from each participant.

### Epidemiological Investigation and Anthropometric Measurements

At the baseline, the participants were interviewed face to face with a structured questionnaire; the details were reported previously (2, 16). The information solicited in the questionnaire included demographic data, such as date of birth, sex, educational level, smoking and alcohol drinking behaviors, physical activity, dietary habits, and family history of diabetes. Original smoking behavior in the cohort was investigated as current, previous, and never. Current smoking was defined as a person’s smoking at least one cigarette per day for (at least) 1 year. Previous smoking was defined as a person’s having quit for at least 1 year. Both current smoking and previous smoking were defined as smoking in the multiple regression. The alcohol consumption in the cohort was measured as the frequency of drinking and was categorized into two groups: ≥3 times/week and <3 times/week. The smoking rate and drinking rate were calculated as the percentage of smokers or drinkers in each group. Information on physical activity was collected with the International Physical Activity Questionnaire (IPAQ) (short vision) (3). According to the instructions, calculate the energy consumed by each activity in units of metabolic equivalent (MET) minutes per week (MET-m/week). The threshold of total physical activity ≥600 MET-m/week is considered to be a moderate or high physical activity level. Calculate the sedentary time per week, and divide the participants into light, moderate, and heavy according to the quartile of sedentary time.

The anthropometric index, which includes weight, height, WC, systolic BP (SBP), and diastolic BP (DBP), were measured by well-trained investigators or doctors with a standard protocol (2). Height and weight were measured when the subjects wore light clothing and without shoes. WC was measured at the midpoint between the iliac crest and lowest rib. The record retains a decimal place. BP was measured in a sitting position with a mercury sphygmomanometer. SBP and DBP were reported as the average of three repeat measurements at 30-s intervals.

The overnight fasting blood samples were collected for each subject. Biochemical variables, including TG, total cholesterol (TC), HDL-C, and low-density lipoprotein cholesterol (LDL-C), were determined by a biochemical autoanalyzer (Hitachi 7060, Tokyo, Japan). FPG was analyzed using the glucose oxidase method with the Beckman Glucose Analyzer (Beckman Instruments, Irvine, CA, USA).

### Definitions of Obesity and Metabolic Abnormality

BMI was calculated as weight (kg) divided by the square of height (m^2^). General obesity was defined by BMI with the criteria of the Working Group on Obesity in China (WGOC) (4). Obesity was operationalized as a BMI ≥28 kg/m^2^, overweight was a BMI ≥24 and <28 kg/m^2^, and normal weight was a BMI ≥18.5 and <24 kg/m^2^. Metabolically abnormal components included 1) high TG (≥1.7 mmol/L), 2) low HDL-C (men <1.03 mmol/L and women <1.29 mmol/L), 3) high SBP (≥130 mmHg) or DBP (≥85 mmHg) or use of antihypertensive drug therapy, and (16) high FPG (≥5.6 mmol/L) or antidiabetic treatment (5). Metabolic abnormality was defined as ≥2 abnormal components, whereas metabolic normality was defined as ≤1 abnormal component.

Therefore, metabolically normal and normal weight (MNNW) represents metabolic normality and normal weight, and MANW represents metabolic abnormality but normal weight. Metabolically abnormal and obesity/overweight (MAO) represents metabolic abnormality and obesity/overweight, whereas MNO represents metabolic normality but obesity/overweight.

### Follow-Up and Case Ascertainment

All the subjects were followed up after the baseline investigation and ended on the date of diagnosis of T2DM, lost to follow-up, or censoring date (December 31, 2017), whichever came first. After exclusion of type 1 diabetes mellitus, gestational diabetes mellitus, or diabetes due to other causes, T2DM was defined using the following criteria: 1) FPG ≥7.0 mmol/l, 2) any treatment for diabetes, and 3) self-reported history of diabetes, which were previously diagnosed by clinical physicians.

In the cohort, the incidence of T2DM was identified through the Zhejiang Chronic Disease Surveillance System or by field-epidemiological investigation. The details of the Zhejiang Chronic Disease Surveillance System were reported previously (15). The investigators also asked each participant if they had ever been diagnosed with T2DM by clinical physicians.

### Statistical Analyses

Continuous variables of normal distribution were described as mean and SDs, while continuous variables of skewed distribution were expressed as medians and interquartile ranges (IQRs). Categorical variables were expressed as number (%).

The chi-square test was used to compare categorical variables such as metabolic components and behavioral factors. One-way ANOVA was used to compare the four metabolic phenotypes.

The person-year of follow-up was calculated from the date of recruitment to the end of the follow-up period. The incidence of T2DM was calculated as the number of new cases divided by the person-years of follow-up. We have tested assumptions of the Cox proportional-hazards model using the Kaplan–Meier survival curves and a graphical approach based on a cumulative risk function, and the study was consistent with the Cox proportional-hazards model assumptions. The hazard ratio (HR) and its 95% CI of the obese phenotypes were calculated using a multiple Cox regression model, with the MNNW group as a reference group. There are three models used in the analysis to adjust for potential confounding factors: unadjusted model 1, model 2 with adjustments for age and sex, and model 3 with adjustments for age, sex, smoking, drinking, physical activity, and family history of diabetes.

In the sensitivity analysis, we first excluded subjects whose outcomes occurred in the first 2 years of follow-up to avoid reverse causation. The analysis was performed using the strict definition of metabolic normality as zero abnormal components, while metabolic abnormality was defined as ≥1 abnormal components. We also analyzed subjects who were restricted to non-smokers or stratified by sex.

A two-tailed *p* < 0.05 was considered statistically significant. All statistical analyses were conducted using PASW Statistics version 20.0 for Windows (SPSS Inc., Chicago, IL, USA).

## Results

### Baseline Characteristics of the Subjects

There were 17,238 participants recruited in this study. Their mean age is 54.1 years (SD: 13.9 years), and 42.6% of the subjects were male.

The basic characteristics of the subjects with the four obesity phenotypes are presented in **Table 1**. The prevalence of MAO, MNO, and MANW among the subjects was 25.8%, 13.4%, and 25.1% (in **Supplementary Table 1**). There were significant differences in anthropometric variables and metabolic components among the subjects with the four obesity phenotypes except for drinking rate (all *p*-values <0.05).

**Table 1:** Baseline characteristics of subjects in the Zhejiang cohorts.

Variables	Zhejiang cohort				
	MNNW	MNO	MANW	MAO	*P*-Value
Number	6,159	2,315	4,321	4,443	
Men (%)	42.3	44.5	41.4	43.2	<0.001
Age (years)	49.3 (14.2)	49.2 (12.3)	60.6 (12.5)	56.8 (12.1)	<0.001
Weight (kg)	55.7 (6.7)	67.7 (8.4)	55.5 (7.0)	68.6 (9.0)	<0.001
BMI (kg/m^2^)	21.4 (1.5)	26.1 (2.0)	21.8 (1.5)	26.7 (2.2)	<0.001
WC (cm)	75.1 (6.8)	85.8 (7.2)	77.7 (6.5)	88.7 (7.3)	<0.001
Smoking rate, n (%)	1,419 (24.5)	519 (23.7)	892 (21.9)	923 (21.9)	0.003
Drinking rate, n (%)	1,504 (27.3)	562 (27.0)	1,045 (27.0)	1,074 (26.5)	0.829
Physical activity					<0.001
Heavy, n (%)	1,094 (19.2)	395 (18.4)	653 (16.2)	706 (16.8)	
Moderate, n (%)	1,096 (19.3)	439 (20.5)	604 (15.0)	621 (14.8)	
Light, n (%)	3,495 (61.5)	1,310 (61.1)	2,780 (68.9)	2,869 (68.4)	
SBP (mmHg)	115.2 (11.6)	118.3 (10.4)	143.3 (20.2)	142.9 (19.7)	<0.001
DBP (mmHg)	70.7 (8.4)	73.6 (7.8)	83.0 (11.2)	85.0 (11.4)	<0.001
TG (mmol/l)	1.1 (0.6)	1.4 (0.8)	1.8 (1.4)	2.2 (1.6)	<0.001
FPG (mmol/l)	4.7 (0.6)	4.8 (0.6)	5.0 (0.7)	5.1 (0.7)	<0.001
HDL-C (mmol/l)	1.7 (6.6)	1.6 (6.6)	1.5 (5.0)	1.5 (8.8)	<0.001

MNNW, metabolically normal weight; MANW, metabolically abnormal but normal weight; MNO, metabolically normal obesity/overweight; MAO, metabolically abnormal obesity/overweight; n (%), count (percentage); BMI, body mass index; WC, waist circumference; SBP, systolic blood pressure; DBP, diastolic blood pressure; TG, triglycerides; FPG, fasting plasma glucose; HDL-C, high-density lipoprotein cholesterol.

### The Risk of Type 2 Diabetes Mellitus in Subjects With Different Metabolic Phenotypes of Obesity/Overweight

**Figure 1:**
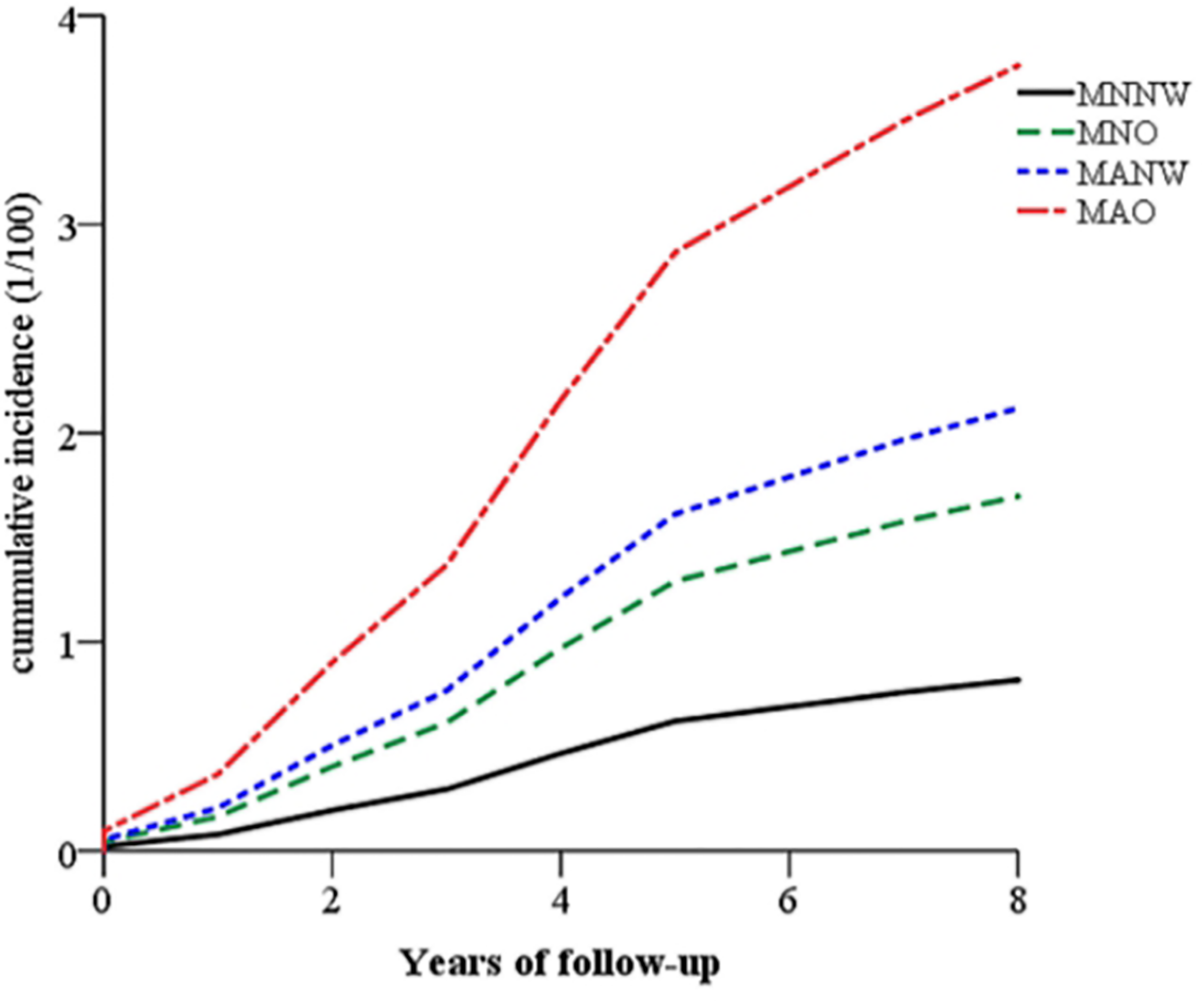
The overall trends of the cumulative incidence of T2DM in the subjects with different phenotypes of obesity after follow-up in the Zhejiang cohort. The subjects with MAO (red and solid line) had the highest incidence of T2DM, followed by MANW (blue and dotted line) and MNO (green and dashed line). MNNW (black and dashed-dotted line) had a lower risk. T2DM, type 2 diabetes mellitus; MAO, metabolically abnormal and obesity/overweight; MANW, metabolically abnormal but normal weight; MNO, metabolically normal but obesity/overweight; MNNW, metabolically normal and normal weight.

In the Zhejiang cohort, 327 new T2DM cases were identified during follow-up. The MNNW subjects had 41,233.0 person-years of follow-up, whereas MNO had 15,705.0 person-years, MANW had 27,945.0 person-years, and MAO had 292,74.0 person-years. The incidence rates of T2DM in the MNNW, MNO, MANW, and MAO were 1.02, 2.23, 3.08, and 5.60 per 1,000 person-years, respectively. The overall trends of T2DM incidence in the subjects with different phenotypes of obesity/overweight are presented in **Figure 1** and **Table 2**. The subjects with MAO had the highest incidence of T2DM, followed by MANW, MNO, and MNNW (**Figure 1**). After adjustment for age, sex, smoking, drinking, physical activity, and family history of diabetes in a multiple Cox regression model, MAO increased the risk for T2DM, with an HR of 4.67 (95% CI: 3.23–6.76) compared with that of the MNNW subjects. MANW and MNO had HRs of 2.61 (95% CI: 1.74–3.92) and 2.09 (95% CI: 1.29–3.38), respectively (**Table 2**). Consistent results were found in model 1 and model 2. In analyzing the subjects with overweight and obesity, subjects with metabolic abnormalities had a risk of T2DM, with HRs of 5.92 (3.77–9.31) for obesity, 4.29 (2.92–6.31) for overweight, and 2.60 (1.73–3.90) for normal weight. The subjects with metabolically normal obesity had risks of 3.08 (1.30–7.30) and 1.93 (1.16–3.23) in metabolically normal overweight subjects. However, no significant interaction was found (*p* > 0.05), and this result may be due to the limited sample size (**Supplementary Table 2**).

**Table 2:** Incidences and hazard ratios for type 2 diabetes mellitus in different obese/overweight phenotypes in the Zhejiang cohorts.

	MNNW	MNO	MANW	MAO
N	6,159	2,315	4,321	4,443
Person years of follow-up	41,233.0	15,705.0	27,945.0	29,274.0
Cases of type 2 diabetes	42	35	86	164
Incidence rate (per 1,000 person years)	1.02	2.23	3.08	5.60
Hazard ratio (95% CI)^§^				
Model 1	Ref	2.20 (1.41–3.45)	3.00 (2.07–4.34)	5.51 (3.92–7.73)
Model 2	Ref	2.21 (1.41–3.46)	2.68 (1.83–3.92)	5.12 (3.63–7.22)
Model 3	Ref	2.09 (1.29–3.38)	2.61 (1.74–3.92)	4.67 (3.23–6.76)

MNNW, metabolically normal and normal weight; MANW, metabolically abnormal but normal weight; MNO, metabolically normal but obesity/overweight; MAO, metabolically abnormal and obesity/overweight.

^§^Model 1: unadjusted Cox model. The overall model significance as calculated using Wald test is p < 0.001.

Model 2: adjusted for age and sex. The overall model significance as calculated using Wald test is p < 0.001.

Model 3: adjusted for age, sex, smoking, drinking, physical activity, and family history of diabetes. The overall model significance as calculated using Wald test is p < 0.001.

### The Correlations Between the Number of Components With Metabolic Abnormalities and the Risk of Type 2 Diabetes Mellitus in Normal-Weight Subjects

**Table 3** shows the correlations between the number of components with metabolic abnormalities and the HRs of T2DM with the normal-weight subjects. Taking subjects with no abnormal component as a reference, all other subjects had an increased risk of T2DM after adjusted for age, sex, smoking, drinking, physical activity, and family history of diabetes. Furthermore, the HRs of T2DM were 2.38 (95% CI: 1.42–4.01) in the group with two abnormalities and 3.92 (95% CI: 2.17–7.10) in the group with three and four abnormalities. The HRs of T2DM were significantly positively correlated with the number of components with metabolic abnormalities (all *p*-values for trends <0.05).

**Table 3:** The correlations between the number of components with metabolic abnormalities and hazard ratios of T2DM in normal-weight subjects.

Number of abnormal metabolic components	HR (95% CI)
0	Ref
1	0.83 (0.48–1.46)
2	2.38 (1.42–4.01)
3+4	3.92 (2.17–7.10)
*p for trend*	<0.001

The HR (95% CI) adjusted for age, sex, smoking, drinking, physical activity, and family history of diabetes.

T2DM, type 2 diabetes mellitus; HR, hazard ratio.

### Sensitivity Analysis

The results of the sensitivity analysis of the study are presented in **Table 4**. Consistent results were found after stratification by sex in males and when using WC as an index of obesity evaluation, and the risk of MNO compared with MNNW was higher than that of MANW. No significant difference was found if metabolically normality was defined as zero abnormalities in metabolic components or restricting the subjects to non-smokers.

**Table 4:** Sensitivity analysis.

	MNNW	MNO	MANW	MAO
Metabolic normality that defined as zero abnormal component	Ref	0.97 (0.39–2.4)	1.98 (1.22–3.2)	4.18 (2.64–6.63)
Subjects without smoking	Ref	1.88 (1.09–3.22)	2.79 (1.79–4.34)	4.00 (2.65–6.05)
WC as index of obesity	Ref	3.95 (1.83–8.50)	2.81 (2.06–3.83)	6.57 (4.12–10.46)
after excluding the first 2 years of follow-up	Ref	1.89 (1.05–3.42)	2.93 (1.82–4.73)	5.06 (3.26–7.86)
Males	Ref	3.57 (1.58–8.05)	3.03 (1.45–6.36)	6.67 (3.39–13.11)
Females	Ref	1.62 (0.87–3.01)	2.70 (1.65–4.44)	4.39 (2.78–6.94)

Adjusted for age, sex, smoking, drinking, physical activity, and family history of disease.

MNNW, metabolically normal and normal weight; MANW, metabolically abnormal but normal weight; MNO, metabolically normal but obesity/overweight; MAO, metabolically abnormal and obesity/overweight; WC, waist circumference.

Qualitatively consistent results were also found when the analysis was performed without adjustment, when adjusting for sex and age only or when making additional adjustments for smoking, drinking, physical activity, and family history of diabetes (**Tables 2**, **3**).

## Discussion

In this prospective cohort study, we found that MANW subjects had a higher T2DM risk than the MNNW subjects. The risk was also positively correlated with the number of abnormal components in normal-weight subjects.

There are different metabolic heterogeneities in obese and normal-weight subjects. Heterogeneous phenotypes may be associated with the risk of long-term effects. Many previous studies have investigated the effects of MNO, but the results have been inconsistent (6–13). These inconsistencies may be due to the different definitions of obesity and metabolic abnormalities, race, region, age, gender, etc. These inconsistencies raised the debate of MNO regarding whether Metabolic health but obesity (MHO) is a health status or a transitional status to MAO (7, 14). In this prospective study with a relatively large sample, we found that MNO increased the risk of T2DM compared with MNNW counterparts but had less risk than MAO participants. These results were consistent with many previous studies (6, 12, 13, 17). From this perspective, MNO was not a healthy status but had a lower risk than that associated with MAO.

Although previous studies have mainly focused on MNO, recently, researchers have started to pay attention to the biological effect of MANW. MANW is an individual with a normal BMI and metabolic abnormalities. This phenotype accounted for approximately 5% to 45% of the normal-weight individuals (18). This prevalence varied based on the definition of MANW, ethnicity, region, age, and gender distributions. In this study, we found that 25.1% of normal-weight individuals were MANW. Compared with their MNNW counterparts, MANW subjects usually have higher levels of visceral and ectopic fat as well as lower lean body mass and insulin sensitivity (19). MANW subjects might have higher risks of long-term effects (17, 20, 21). Reliable evidence is still lacking from prospective studies with large sample sizes, especially from Asian populations, including Chinese populations. Chinese people usually have a low BMI and fat-free mass and higher body fat and visceral fat (19), and they have more abnormal profiles of metabolic components with the same BMI (22, 23). Therefore, the effects of heterogeneous phenotypes might be different between Asian and Western populations. In this study, we found that MANW subjects had approximately three times the T2DM risk compared with MNNW subjects (HR = 2.61, 95% CI: 1.74–3.92) in model 3. Furthermore, MANW subjects also had a higher T2DM risk than their MNO counterparts when compared with MNNW.

In the cohort, the subjects with obesity and abnormalities also had the highest risks (HR = 5.92, 95% CI: 3.77–9.31) (in **Supplementary Table 2**); however, their interaction did not reach statistical significance. This negative association might be due to the limited sample size. In addition to adverse metabolic effects, obesity has multiple other effects that might increase T2DM risk. On the other hand, other risk factors induce metabolic abnormalities in addition to obesity. Therefore, a synergistic effect may be yielded once obesity and metabolic abnormalities are combined. In normal-weight subjects, the number of abnormal components significantly correlated with the HRs of T2DM. Integrating these results, metabolic status seems more important than obesity. This finding was consistent with the result of Liu’s study (13).

These results indicated that MANW individuals were also the higher-risk population for T2DM. Compared with individuals with MNO and MAO, MANW more easily masks the need for screening, thereby delaying diagnosis and treatment and neglecting health management due to seemingly normal weight or normal BMI. Therefore, the public health implication in this study lies in the fact that phenotype-based management is important for obesity and that MANW individuals should be given more attention in clinical and public practice. Our results showed that the average age of the MANW group was higher than that of the other three groups and that in addition to weight control, attention should be paid to changes in biochemical indicators such as blood glucose, BP, HDL-C, and TG in the MANW (24), because in these people with normal BMI, it is easy to ignore the detection of these indicators. Annual health checkups and the modification of dietary habits are necessary. For example, improving the diet such as a diet rich in fruits and vegetables can reduce BP (25), and modifying macronutrient composition can affect lipid levels (26).

The Zhejiang cohort was recruited with a relatively large sample size of 17,238 participants and a long follow-up period of 10 years. The epidemiological data and the biochemical measurements were collected by trained health professionals following standard protocols. The incidences of T2DM were ascertained by record linkage with the data from the local chronic disease surveillance system or by field-epidemiological investigation. The potential confounding bias was controlled with a multiple Cox regression model, although similar results were found using different models. Sensitivity analysis also indicated consistent results under different conditions. These strengths increased the reliability of the evidence.

However, there were some limitations in the study. First, there were multiple definitions of obesity and metabolic abnormalities, and no consensus has been reached until now, so the results may restrict the extrapolation to some previous studies using different definitions. To increase extrapolation, the present study used the most common definition of metabolic abnormality of ≥2 abnormal metabolic components. Second, repeated baseline surveys are ongoing, and the dynamic information on metabolic and obesity status has not been completely collected.

## Conclusion

Briefly, we found that MANW subjects had a higher T2DM risk, and this risk was higher than that in MNO subjects when compared with MNNW. The risk positively correlated with the number of abnormal components in normal-weight subjects. Phenotype-based management is important for obesity, and MANW individuals should be given more attention in clinical and public practice.

## Data Availability Statement

The datasets used and/or analyzed in the current study are available from the corresponding authors and investigating coordinators upon reasonable request.

## Ethics Statement

The studies involving human participants were reviewed and approved by The Ethics Committees of Zhejiang University School of Medicine. The patients/participants provided their written informed consent to participate in this study.

## Author Contributions

All authors listed have made substantial, direct, and intellectual contribution to the work and approved it for publication.

## Funding

This work was supported by grants from the National Key Research and Development Program of China (2017YFC0907004), the Hangzhou Science and Technology Project (20171226Y27), and Zhejiang Health Science and Technology Project (2021KY268).

## Conflict of Interest

The authors declare that the research was conducted in the absence of any commercial or financial relationships that could be construed as a potential conflict of interest.

## Publisher’s Note

All claims expressed in this article are solely those of the authors and do not necessarily represent those of their affiliated organizations, or those of the publisher, the editors and the reviewers. Any product that may be evaluated in this article, or claim that may be made by its manufacturer, is not guaranteed or endorsed by the publisher.

## Abbreviations

MNNW, metabolically normal and normal weight; MANW, metabolically abnormal but normal weight; MNO, metabolically normal but obesity/overweight; MAO, metabolically abnormal and obesity/overweight; BMI, body mass index; WC, waist circumference; TG, triglycerides; FPG, fasting plasma glucose; HDL-C, high-density lipoprotein cholesterol; SBP, systolic blood pressure; DBP, diastolic blood pressure; LDL-C, low-density lipoprotein cholesterol; WHR, waist–hip ratio; WHtR, waist–height ratio.
