# Supplementary material for: Metabolically Abnormal But Normal-Weight Individuals Had a Higher Risk of Type 2 Diabetes Mellitus in a Cohort Study of a Chinese Population
Source: Front Endocrinol (Lausanne). 2021 Nov 3;12:724873. doi: 10.3389/fendo.2021.724873 (PMC8595277; doi:10.3389/fendo.2021.724873)
Supplement: Supplementary file 1 [file Table_1.docx]

Supplemental table 1. Baseline characteristics of subjects in Zhejiang cohorts

|  | Zhejiang cohort |
| --- | --- |
| Number | 17238 |
| Men (%) | 42.6 |
| Age (years) | 54.1(13.9) |
| BMI (kg/$m^{2}$) | 23.5(3.0) |
| WC (cm) | 80.7(9.0) |
| Smoking rate (%) | 23.1 |
| Drinking rate (%) | 27.0 |
| Physical activity |  |
| Heavy (%) | 17.7 |
| Moderate (%) | 17.2 |
| Light (%) | 65.1 |
| SBP (mmHg) | 129.8(21.2) |
| DBP (mmHg) | 77.9(11.8) |
| TG (mmol/l) | 1.6(1.2) |
| FPG (mmol/l) | 4.9(0.7) |
| HDL-C (mmol/l) | 1.6(6.9) |
| Obesity phenotypes |  |
| MNNW (n, %) | 6159(35.79) |
| MANW (n, %) | 4321(25.1) |
| MNO (n, %) | 2315(13.4) |
| MAO (n, %) | 4443(25.8) |

Data are presented as mean (standard deviation) for quantitative data and percentage for qualitative data.

BMI, body mass index; WC, waist circumference; SBP, systolic blood pressure; DBP, diastolic pressure; TG, triglycerides; FPG: Fasting plasma glucose; HDL-C, high-density lipoprotein cholesterol.

MNNW, metabolically normal and normal weight. MANW, metabolically abnormal but normal weight. MNO, metabolically normal but obesity/overweight. MAO, metabolically abnormal and obesity/ overweight.

Supplemental table 2. The hazard ratios^#^ for type 2 diabetes mellitus in different obese phenotypes categorized by obesity and overweight

| Metabolic status | Normal weight | overweight | obesity | P interaction |
| --- | --- | --- | --- | --- |
| normality | 1(reference) | 1.93(1.16-3.23) | 3.08(1.30-7.30) | > 0.05 |
| abnormality | 2.60(1.73-3.90) | 4.29(2.92-6.31) | 5.92(3.77-9.31) |  |

#,Adjusted for age, sex, smoking, drinking, physical activity, and family history of diseases.
